# Comparison of the Physicochemical Properties of Carboxylic and Phosphonic Acid Self-Assembled Monolayers Created on a Ti-6Al-4V Substrate

**DOI:** 10.3390/ma13225137

**Published:** 2020-11-14

**Authors:** Michal Cichomski, Milena Prowizor, Dorota Anna Kowalczyk, Andrzej Sikora, Damian Batory, Mariusz Dudek

**Affiliations:** 1 Department of Materials Technology and Chemistry, University of Lodz, Faculty of Chemistry, Pomorska 163, 90-236 Lodz, Poland; milena.prowizor@chemia.uni.lodz.pl; 2 Department of Solid State Physics, Faculty of Physics and Applied Informatics, University of Lodz, Pomorska 149/153, 90-236 Lodz, Poland; dorota.kowalczyk@uni.lodz.pl; 3 Department of Nanometrology, Faculty of Microsystem Electronics and Photonics, Wroclaw University of Science and Technology, Janiszewskiego 11/17, 50-372 Wrocław, Poland; andrzej.sikora@pwr.edu.pl; 4 Department of Vehicles and Fundamentals in Machine Design, Lodz University of Technology, Stefanowskiego 1/15, 90-924 Lodz, Poland; damian.batory@p.lodz.pl; 5 Institute of Materials Science and Engineering, Lodz University of Technology, Stefanowskiego 1/15, 90-924 Lodz, Poland; mariusz.dudek@p.lodz.pl

**Keywords:** carboxylic/phosphonic acid, Ti-6Al-4V alloy, adhesion, friction, nano-/microtribology

## Abstract

This study compared the tribological properties in nano- and millinewton load ranges of Ti‑6Al-4V surfaces that were modified using self-assembled monolayers (SAMs) of carboxylic and phosphonic acids. The effectiveness of the creation of SAMs with the use of the liquid phase deposition (LPD) technique was monitored by the contact angle measurement, the surface free energy (SFE) calculation, X-ray photoelectron spectroscopy (XPS), and Fourier-transform infrared spectroscopy (FTIR) measurements. The obtained results indicated that more stable and well-ordered layers, which were characterized by the lowest values of the coefficient of friction, adhesion, and wear rate, were obtained using phosphonic acid as a surface modifier. Based on the obtained results, it was found that the Ti-6Al-4V alloy modified by phosphonic acid would be the most advantageous for practical applications, especially in micro- and nanoelectromechanical systems (MEMS/NEMS).

## 1. Introduction

Titanium and its alloys are widely used in various important fields of industry in which their physicochemical and mechanical properties play a very important role [1,2]. The high strength-to-weight ratio and low density of the titanium alloy Ti-6Al-4V make it a very good candidate to be used in aerospace and automotive engineering, chemical plants, power generation, and surgery and medicine [3,4,5]. The main benefits of using titanium alloys in biomedical applications for implants and prostheses are their Young’s modulus, which is similar to the bone, their biocompatibility, and their excellent corrosion resistance [6,7]. 

Despite numerous advantages, titanium alloys also have a number of disadvantages, which scientists are trying to eliminate. The major drawback to the use of Ti-6Al-4V is the presence of toxic elements, such as aluminum (Al) and vanadium (V). These ions are released to the surrounding tissues when the alloy is implanted into the human body [8,9,10]. Another inconvenience associated with the engineering applications of Ti-6Al-4V alloys is their poor tribological characteristics [9]. To eliminate these negative effects, numerous coating technologies, ion implantation techniques, shot peening, or thermal oxidation techniques can be used. These processes enable changing the nature of the surface via modification of the top layer of a titanium alloy and, at the same time, improving their mechanical, chemical, biological, and tribological properties [7,11,12,13,14,15]. 

An interesting option for modification/treatment of a Ti-6Al-4V alloy surface can be an application of self-assembled monolayers (SAMs). The creation of a self-assembled monolayer on the surface of a Ti-6Al-4V alloy is a way to change the properties of the tested material that is relatively quick, simple, and does not require high production costs. It is also a kind of barrier between the material and the human body, which is extremely important from a biomedical point of view. Additionally, by using this method of modification, it is possible to control the surface composition, as well as to create an oriented, well-ordered, and stable layer [16]. Many studies also show the positive effect of the created layer on the so-called interfacial properties of the surface (wettability, ion exchange, or resistance to UV light) [17,18]. These properties can be easily adjusted by selecting an appropriate functional group for the modifying compound. Among the large group of commonly used modifiers, phosphonic or carboxylic acids are becoming more and more popular [18,19,20]. It was found that these acids form well-defined and stable layers with a range of metal oxide surfaces; furthermore, they do not polymerize like silanes and are not sensitive to humidity changes. In their study, Krzykawska et al. [21] compared both the order and formation of SAMs of the carboxylic acid with commonly used and well-known SAMs of thiol on the metal substrate. These studies have shown that carboxylic acid SAMs are an interesting alternative to the ones used so far, namely, thiol monolayers, because they are characterized by high-quality ordering, which has a significant impact on the physicochemical properties of the surface [21]. In turn, the information available in the literature on the stability of phosphonate SAMs on Al, Ti, or Zr indicate high bond durability under various conditions, for example, a wide range of pH, temperature, and humidity, which is important from the point of view of their potential applications [18,22,23,24]. 

In this work, tribological properties of carboxylic acid (PFDA) SAMs and phosphonic acid (PFDPA) SAMs measured at the micro and nano scales using a microtribometer T-23 and atomic force microscopy (AFM) were compared. The SAMs were created on a Ti-6Al-4V surface using a liquid phase deposition (LPD) technique. The tribological properties of SAMs were studied in reference to their physicochemical properties, which were characterized using contact angle measurements, Fourier-transform infrared (FTIR) spectroscopy, and X-ray photoelectron spectroscopy (XPS). In this study, for the first time, the influence of a carboxylate and phosphonate layer on the surface properties of a Ti-6Al-4V alloy in nanoscale was specified.

## 2. Materials and Methods 

### 2.1. Materials and Modification Procedures

**Figure 1:**
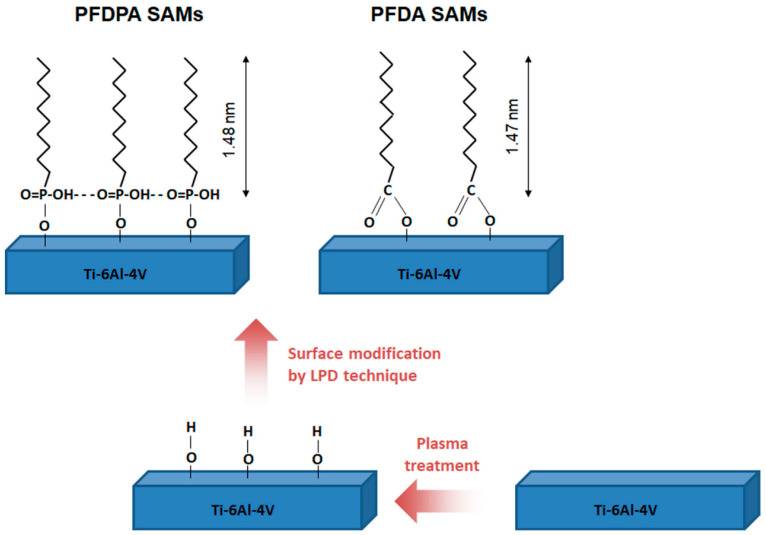
Schematic illustration of the modification processes leading to the creation of 2H,2H,3H,3H-perfluoroundecanoic acid (PFDA) and 1H,1H,2H,2H-perfluorodecylphosphonic acid (PFDPA) self-assembled monolayers (SAMs) on Ti-6Al-4V surfaces. LPD: liquid phase deposition.

The carboxylic and phosphonic acid self-assembled monolayers were created on a titanium alloy (Ti-6Al-4V) surface prepared using the magnetron sputtering technique. A thin coating (100 ± 2 nm) of the Ti-6Al-4V was deposited on Si (100) substrates. Prior to the creation of the SAMs, the Ti-6Al-4V surface was cleaned using radiofrequency oxygen plasma for 15 min in order to oxidize the surface through creating hydroxyl groups, which act as active sites for the formation of bonds between the surface and the modifier. The 1H,1H,2H,2H-perfluorodecylphosphonic acid (PFDPA), 2H,2H,3H,3H-perfluoroundecanoic acid (PFDA) were purchased from ABCR, GmbH & Co. KG, Karlsruhe, Germany. The chemical structures of both modifiers are presented in Figure 1.

### 2.2. Surface Characterization 

The chemical composition of the Ti-6Al-4V surface covered with a thin carboxylate and phosphonate layer was examined by using XPS and FTIR. The XPS analysis was performed using an Omicron ultrahigh vacuum (UHV) system (base pressure below 5 × 10^−8^ Pa). The measurements employing a non-monochromatic Mg Kα_1,2_ source operated at 75 W were obtained. The two-point correction of the energy scale of the instrument based on Au 4f_7/2_ (83.95 eV) and Ag 3d_5/2_ (368.22 eV) lines was applied prior to the measurements. The acquired spectra did not undergo further calibration.

FTIR spectra of the Ti-6Al-4V substrate was collected before and after modification using a Nicolet iS50 spectrometer (Thermo Fisher Scientific, Waltham, MA, USA)with a grazing angle attenuated total reflectance (GATR) accessory from Harrick Scientific Products Inc. with an linearized mercury-cadmium-telluride (MCT) detector. All spectra were registered using 64 scans and at a 4 cm^−1^ resolution in a dry air atmosphere.

The wettability of the surfaces was measured in the laboratory atmosphere at a relative humidity of 45 ± 5% and temperature of 22 ± 2 °C using a DSA-25 Drop Shape Analysis System (KRÜSS GmbH). Three liquids, namely, deionized water (POCH S.A., Gliwice, Poland), glycerine (POCH S.A., Gliwice, Poland), and diiodomethane droplets (POCH S.A., Gliwice, Poland), were used for making contact angle measurements. Drops with a volume of 2 μL were deposited on the surfaces with the use of an automatic syringe. The static contact angle values were reported for five distinct drops deposited on each sample. Next, the surface free energy was calculated using the Van Oss–Chaudhury–Good method. 

AFM microscopy was used to study the topography, adhesive force, coefficient of friction, and wear of the studied surfaces at the nanoscale. The AFM studies were carried out using a Solver P47 apparatus (NT-MDT, Moscow, Russia) operating in air under ambient conditions. Surface topography measurements were carried out in a semicontact mode with a scanned area of 10 × 10 μm and a scan rate of 0.5 Hz. The root-mean-square (RMS) roughness parameter was calculated from the AFM images. Friction and adhesive forces were investigated using a rectangular cantilever made of Si_3_N_4_ with a spring constant k = 0.65 ± 0.07 N/m. The radius of the curvature of the tip was less than 20 nm. The adhesive force was obtained from the force–distance curve as a calculation of the pull-off force. Obtained data from different points on the surface were then averaged from ten force–distance curves. The friction force was calibrated using the method described by Sikora et al. [25]. The coefficient of friction was found using the slope of the friction force versus normal force plots. The following technical parameters of measurements were used: applied load ranged from 5 to 100 nN, scan rate of 1 Hz, and scan size of 10 × 10 μm. No traces of the surface modification due to the test were noticed. Each measurement was repeated three times in different places on the sample surface and the results were averaged. Wear tests on each surface were conducted in contact mode by using a diamond tip with a scan area of 2 × 2 μm with a normal load of 1 µN. 

The microtribological behavior of all studied surfaces was investigated using a T-23 microtribometer. The Si_3_N_4_ ball that was 5 mm in diameter and had a roughness of 5.5 ± 0.5 nm was used as the counterbody sliding over the Ti-6Al-4V surface with a velocity of 25 mm/min and a normal load from 30 to 80 mN. The measurements consisted of six cycles, and after each cycle, the load was increased by 10 mN. All measurements were performed in normal ambient conditions (temperature and humidity) and repeated in three different locations on the surface.

## 3. Results and Discussion

### 3.1. Optimization of the Liquid Phase Deposition Method Parameters

The quality of the self-assembled monolayers produced using the LPD method is influenced by many factors, such as the type of solvent, solution concentration, deposition time, temperature, and pressure of the conducted process, as well as aging of the solution [26,27,28,29]. Analysis of the literature has shown that the most commonly used solvent in this type of modification is ethyl alcohol, while the process is conducted in normal temperature and atmospheric pressure conditions [14]. In our study, those parameters were retained, and the deposition time and concentration of PFDPA and PFDA in the solution of ethyl alcohol were optimized. Based on the measured contact angle, the optimal deposition time was selected for different solution concentrations (from 0.01 to 0.1 wt.%). Before the LPD processes, the surface of the Ti-6Al-4V substrate was treated using oxygen plasma for 15 min. After this process, the surface was completely hydrophilic, where the contact angle measured immediately afterward was about 5 degrees (for an untreated surface, the angle was 71°, which was found using the tests as a reference sample). 

**Figure 2:**
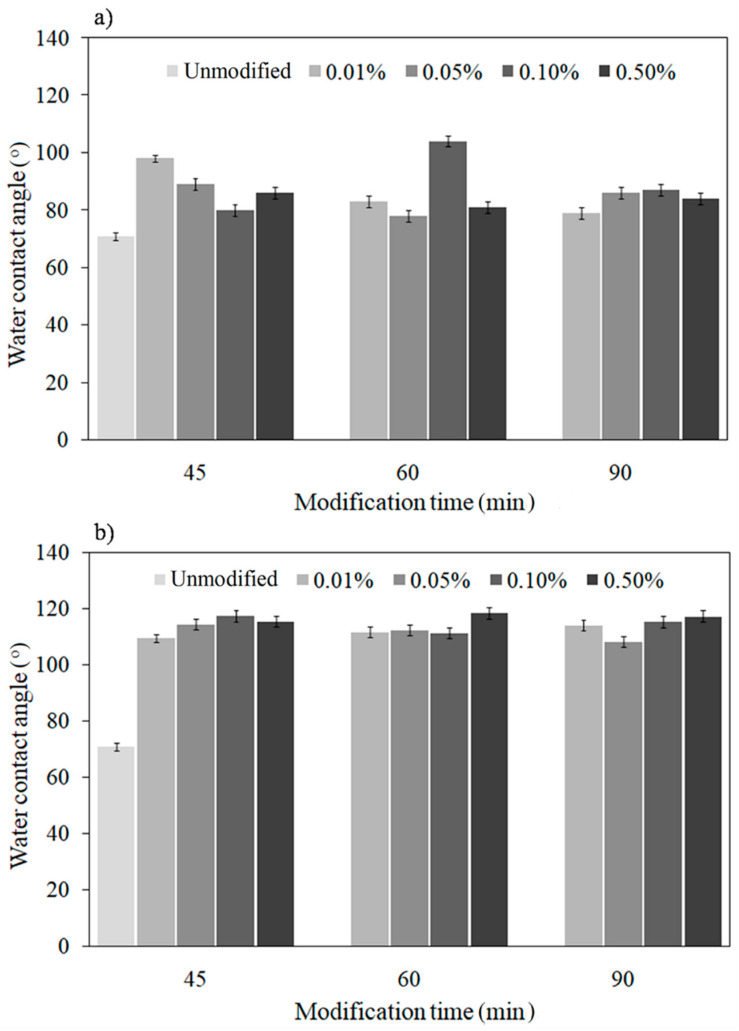
Water contact angle values for different deposition parameters of (**a**) PFDA and (**b**) PFDPA.

Figure 2 shows the results of the water contact angle analysis for the PFDPA and PFDA layers created on oxygen-plasma-treated Ti-6Al-4V surfaces (the measurements took place immediately after 24 h of a post-creation annealing process). By analyzing the obtained results, it was found that for PFDA SAMs (Figure 2a), the most hydrophobic surface (104°) was observed for a solution concentration equal to 0.10 wt.%. For the PFDPA layers (Figure 2b), the highest water contact angle (118°) was achieved for a 0.50 wt.% solution concentration. In the case of both compounds, the most hydrophobic surface was obtained after 60 min of deposition time, thus indicating that this time was optimal for the creation of SAMs. 

Comparing the wetting properties of a titanium alloy surface before (71°) and after modification, it can be seen that the SAMs layers improved the surface hydrophobicity. This was primarily caused by the presence of CF_3_-terminated films formed out of the well-ordered layer on the top of the surface [30,31,32]. The –CF_3_ group is non-polar, thus it repels water molecules from its surface, striving for the smallest possible contact area. Due to differences in the electronegativity of atoms in a C–F bond, the trifluoromethyl group is a strong dipole, which causes changes in the wettability [33,34,35,36,37]. Moreover, as is already widely known, fluorine is characterized by a high electronegativity (the most electronegative of all elements). Its negative charge repels the positive charge of the water molecule, and as a consequence, reduces water’s access to the surface. It also has a high ionization potential and very low polarizability. This last parameter has a significant influence on the increase in the hydrophobicity [33]. 

The hydrophobic properties of the titanium alloy surface after modification by the PFDPA compound were higher in comparison to those obtained for PFDA (Figure 2). This was due to the structure of the modifier and the method of forming monolayers on the substrate. The phosphonic acids used for modification contain the same –CF_3_ functional groups and the same carbon chain length (perfluorodecyl) but different kinds of head groups. The PFDA molecule forms substrate–O–C bonds, while the PFDA molecule creates substrate–O–P bonds. In the case of PFDPA, each molecule in the monolayer is covalently attached to the surface. The layer growth is random, and in this case, the van der Waals interaction between the molecules is less important than the interaction between the molecules and the surface [38]. In the case of PFDA, the presence of a –COO^−^ ionic group in the molecule enables the chemisorption between the carboxylic acid head group and the oxide surface. It can be concluded that a covalent bond on the oxidized Ti-6Al-4V substrate surface is formed similarly to the case of the reaction of the carboxylic acid with oxidized Al [39,40].

The difference in wettability between both deposited layers is apparently due to the differing strengths of the chemisorption of the molecule head groups of modifiers. Moreover, the presence of P in the head group of PFDPA and C in the head group of PFDA (completely perfluorinated alkyl chain) causes a difference in acidity between PFDA and PFDPA. A difference in acidity correlates with the difference in packing and a high degree of order in the layers of the SAMs [40]. This is why the more close packing layer of Ti-6Al-4V/PFDPA in comparison with Ti-6Al-4V/PFDA exhibited a lower wettability.

### 3.2. Surface Characterization Using XPS and FTIR Measurements

**Figure 3:**
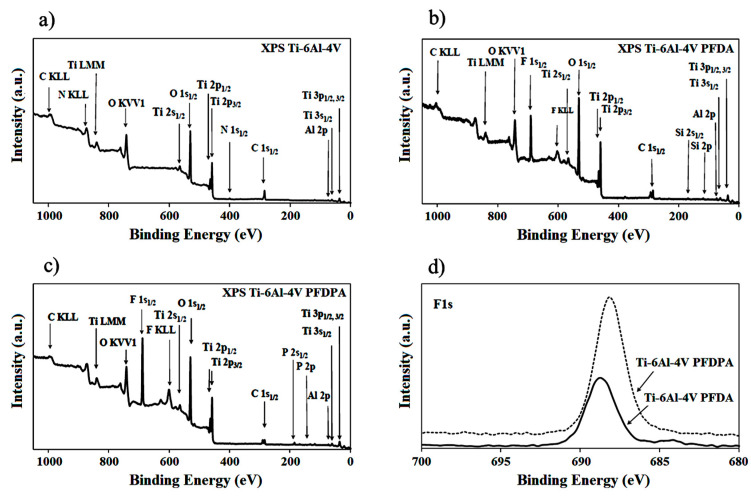
XPS spectra of the Ti-6Al-4V surface before and after modification: (**a**) Ti-6Al-4V substrate, (**b**) PFDA modification, (**c**) PFDPA modification, and (**d**) F 1s_1/2_ peak of fluorine identified in both SAMs.

In order to characterize the surface chemistry and the quality of the modification, spectroscopic techniques were used. In the present study, XPS spectra were recorded for both modified and unmodified Ti-6Al-4V surfaces (Figure 3). The peaks attributed to substrate Ti 2s_1/2_, Ti 3s_1/2_, Ti 2p_1/2_, Ti 2p_3/2_, Ti 3p_1/2_, Ti 3p_3/2_, and Ti LMM can be observed on the XPS spectra before and after modification via both compounds. Moreover, for the unmodified Ti-6Al-4V, the presence of O 1s_1/2_ and O KVV1 peaks is visible too. Their appearance confirmed the presence of oxidized forms on titanium, which is also reported in the literature [41,42]. Due to their presence, this shows that it is possible to carry out chemical modification by using phosphonic acids. The XPS data of Ti-6Al-4V after modification showed signals that are characteristic of phosphorus, namely, P 2s_1/2_ and 2p, which proved that the chemical bond between the molecules and the substrate surface was formed. Moreover, in Figure 3b,c, F 1s_1/2_ and F KLL peaks were observed, which are characteristic for fluorine. The appearance of these peaks confirmed the occurrence of PFDPA and PFDA on the substrate after the deposition process. Furthermore, the difference in the intensity of O 1s_1/2_ and O KVV1 peaks for PFDPA and PFDA spectra was also observed, which was connected with their degree of ordering. The PFDPA SAMs were characterized as being of high quality with close packing and a high degree of order, as the intensity of the oxygen peak on the XPS spectrum was lower.

To verify the quality of the phosphonic layers, high-resolution XPS characterization was also performed. Considering the intensity of the F 1s peaks for both molecules at about 689 eV (Figure 3d) and their peak height ratio, we concluded that the packing of molecules associated with the surface of titanium in the case of PFDPA was better organized than for PFDA.

**Figure 4:**
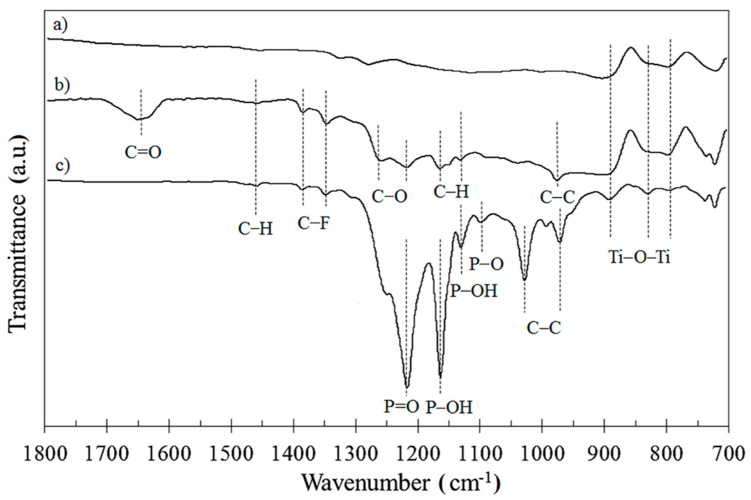
FTIR measurement for (**a**) Ti-6Al-4V, (**b**) Ti-6Al-4V/PFDA, and (**c**) Ti-6Al-4V/PFDPA.

In order to extend the chemical characterization of the modified Ti-6Al-4V substrate and determine how the phosphonic acid and carboxylic acid molecules were bound to the substrate, the FTIR technique was also applied. In the present study, the FTIR spectra were recorded for both modified and unmodified Ti-6Al-4V surfaces, where they were cleaned using radiofrequency oxygen plasma for 15 min in order to oxidize the surface through creating the hydroxyl groups, which acted as active sites for the formation of bonds between the surface and the modifier (Figure 4). On the FTIR spectrum after modification, it peaks in the range of 1350–1386 cm^−1^ were observed, which corresponded to asymmetric and symmetric stretches of C–F for –CF_2_ and –CF_3_ groups occurring in the PFDA and PFDPA molecules. Peaks at 1130, 1162, and 1453 cm^−1^ for PFDA were due to C–H asymmetric stretches. The peak at 980 cm^−1^ is characteristic of the C–C bond, which occurred in the carbon skeleton of the PFDA and PFDPA compounds. Moreover, the peaks at 798, 829, and 899 cm^−1^ were assigned to Ti–O–Ti bond vibrations and were present for the unmodified (higher intensity of peaks) and modified surfaces. 

In the literature, DeRose et al. [40] proposed two ways of bonding PFDA to the surface of aluminum, namely, by forming monodentate or bidentate bonds. For a molecule with a longer carbon chain (such as PFDA or PFDPA), the monodentate bonding scheme is most commonly found. Moreover, Ting et al. [43,44] observed that monodentate binding for carboxylic SAMs of stearic acid on metal oxides is revealed by the presence of the carbonyl C=O stretch at approximately 1740 cm^−1^. For alloys modified by PFDA, the peak originating from the carbonyl C=O group was registered in the region between 1615–1700 cm^−1^, which means that the molecule of PFDA formed monodentate binding with the Ti-6Al-4V surface. 

**Figure 5:**
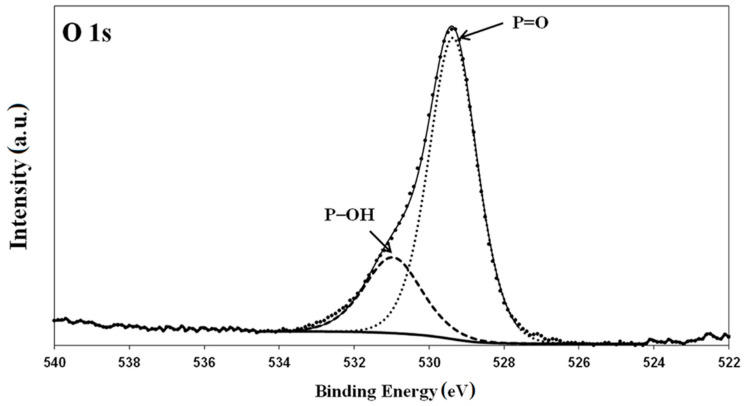
High-resolution XPS spectra of the O 1s peak (with deconvolution) obtained for PFDPA SAMs on a Ti-6Al-4V substrate.

In the case of phosphonic acid (PFDPA), Brodard-Severac et al. [45] indicated that the presence of three oxygen atoms allows for mono-, bi-, and tri-dentate binding with the metal surface. In the case of mono- and bi-dentate binding, the remaining groups (P–OH and P=O) most likely create hydrogen bonds with neighboring phosphonate compounds. Their presence is important because hydrogen-bonding interactions cause stabilization of the monolayer. In this study, for the surface modified by PFDPA, the P=O stretching vibration was manifested as an intense peak on the FTIR spectrum at 1212 cm^−1^. Therefore, the IR characterization suggests that phosphonic acid cannot form tridentate binding with a surface of Ti-6Al-4V. The bands at 1126 and 1160 cm^−1^ corresponded to P–OH stretching vibrations, whereas the peak characteristic for the P–O vibrations was clearly visible around 1100 cm^−1^. This was also confirmed by the XPS analysis. The high-resolution O 1s XPS spectra obtained for the PFDPA SAMs on the Ti-6Al-4V substrate are shown in Figure 5. In the literature, the binding energy values for P=O and P–O groups in phosphonic acids are 530.8–535.0 eV and 529.4–533.6 eV, respectively, whereas the separation between the components is fixed at approximately 2 eV [46,47,48]. The presence of both peaks in the spectrum and relatively low intensity of the P–OH peak indicated that the phosphonate was bound to the oxide surface through monodentate binding [44,49]. In turn, the high intensity of the peak derived from the P=O groups shows that it did not take part in the formation of binding to the Ti-6Al-4V surface. In Figure 5, two peaks derived from the P–OH and P=O bonds were clearly visible. Moreover, the high intensity of the P=O peak indicated that this group did not participate in the bond formation with the alloy surface, which excluded the formation of a tridentate bond. Moreover, the presence of a peak derived from the P–OH bonds in the XPS spectrum indicated that it was also not involved in the formation of a bond between the compound and the surface. On this basis, it can be concluded that the monodentate bond was produced by the modification of Ti-6Al-4V with PFDP acid.

### 3.3. Surface Free Energy Measurements

As it is well known, the wetting properties of a solid surface are generally determined by static and dynamic contact angle measurements and also by a subsequent calculation of the surface free energy (SFE). Several individual methods can be used for the calculation of the SFE, such as Owens, Wendt, Rabel and Kaelble (OWRK), Fowkes, Owens–Wendt, Zisman, and Neumann. However, each of these is based on the commonly known Young’s equation, which states that the value of the SFE is the sum of the interfacial solid/liquid free energy, the product of the surface free energy (surface tension) of a liquid, and cosθ where θ is the contact angle of the liquid droplet [50].

In the present work, the SFE was calculated using the van Oss, Chaudhury, and Good method [51], which provides more information about the examined interactions, especially about the interfacial acid–base and van der Waals interactions. In this theory, the SFE is the sum of the γ^LW^ component based on Lifshitz–van der Waals interactions and the acid–base component γ^AB^ based especially on hydrogen bonding interactions. As depicted in Equation (1), the acid–base term consists of the asymmetric components γ^+^ for the acid interaction and γ^−^ for the base interaction. The ‘‘acid’’ component is also called the acceptor because it is positively charged, thus it is ready to accept electrons; the ‘‘base’’ component is also called the donor because it is negatively charged, thus has extra electrons to give away.

γ^AB^ = 2(γ^+^γ^−^)^1/2^ (1)

Taking Young’s equation: γ_lv_cos θ = γ_sv_ − γ_sl_ into consideration (where θ is the measured contact angle and γ_lv_, γ_sv_, and γ_sl_ are the liquid–vapor, solid–vapor, and solid–liquid interfacial tensions, respectively), the LW/acid–base equation can be written as follows:

γ_l_(1+cos θ) = 2(γ_l_^LW^γ_s_^LW^)^1/2^ + 2(γ_l_^+^γ_s_^−^)^1/2^ + 2(γ_l_^−^γ_s_^+^)^1/2^. (2)

Equation (2) can be used to determine the three surface tension components (γ_s_^LW^, γ_s_^−^, γ_s_^+^) by measuring the contact angles and solving the system of three equations [38].

The surface free energy was calculated for the selected, most effective modifications (0.1 wt.% solution of PFDA and 0.5 wt.% solution of PFDPA with 60 min of deposition time) and the results are presented in Table 1.

The SFE for the unmodified Ti-6Al-4V surface was 47 ± 2 mJ/m^2^, but after the modification, its value significantly decreased. The surface energies of Ti-6Al-4V/PFDPA and Ti-6Al-4V/PFDA were 9.6 ± 0.3 mJ/m^2^ and 21.0 ± 0.3 mJ/m^2^, respectively. Low values of SFE are associated with the presence of non-polar molecules of phosphonic acids on the Ti-6Al-4V surface, which repel a polar drop of water, preventing its contact with the Ti-6Al-4V alloy surface. Comparing both compounds, namely, PFDPA and PFDA, slightly lower surface energy was found for the surface modified by PFDPA, which explains the highest water static contact angle obtained for this sample. The non-polar compound (PFDPA) was characterized by small values of the acid–base and Lifshitz–van der Waals components, and as a consequence, had a high hydrophobicity. The obtained results of the energies show that the γ^LW^ component had the greatest impact on the SFE value. For PFDA, the component based on Lifshitz–van der Waals interactions was 19.9 mJ/m^2^. This value was significantly higher in comparison with the γ^LW^ component for PFDPA. Moreover, the calculated values of γ^ΑΒ^ were very low in comparison to the values of γ^LW^ for both surfaces after modification. As mentioned earlier, this was associated with the hydrophobic, well-ordered layer, which covered the polar groups on the unmodified surface.

**Figure 6:**
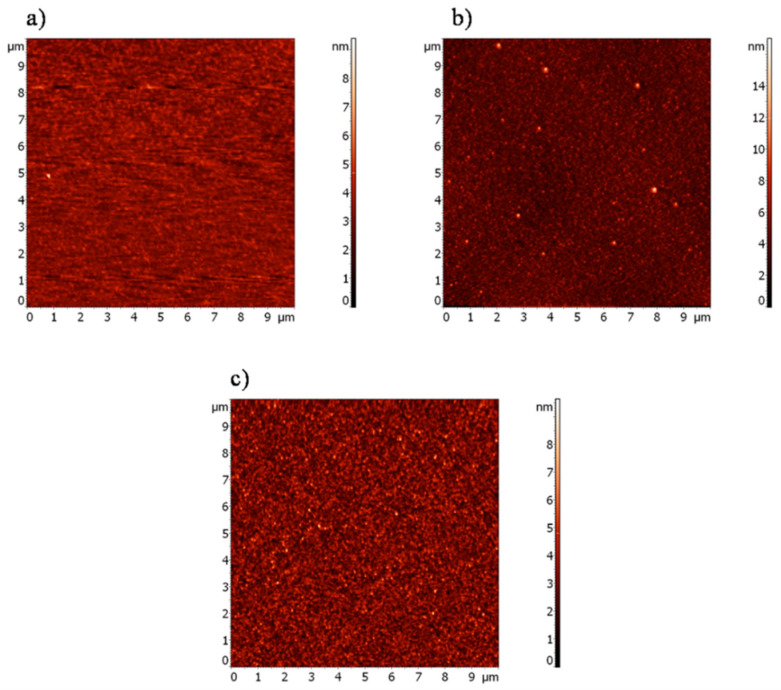
AFM images of (**a**) Ti-6Al-4V, (**b**) Ti-6Al-4V/PFDPA, and (**c**) Ti-6Al-4V/PFDA surfaces.

Figure 6 shows the 2D maps from the AFM measurements of the Ti-6Al-4V surface before and after the creation of the SAMs. The estimated values of the RMS roughness (S_q_) indicated that after the modification, its RMS roughness increased from 0.55 nm to 0.61 nm and 1.01 nm for PFDA and PFDPA, respectively. The AFM data showed that for the carboxylate layers, the roughness was slightly higher than for the pure Ti-6Al-4V surface. This was related to the orientation or arrangement of the chains of the modifier on the surface. The literature confirms that as a result of the acid–base reaction of the carboxylate group with the oxidized metal surface, the perfluoroalkyl chains are arranged in a canted configuration, which increases the surface roughness [39]. The obtained surface roughness data for the Ti-6Al-4V/PFDPA sample also suggests that the phosphonate layer had an impact on the surface roughness. This increase in roughness for Ti-6Al-4V/PFDPA could be due to the formation of agglomerates on the surface, which can be seen in Figure 6. These data show that the highest surface roughness exhibited the most hydrophobic SAMs, which confirmed the observation that the surface roughness affected the contact angle values, and as a consequence, the surface free energy. 

### 3.4. Micro- and Nanotribological Characterization

**Figure 7:**
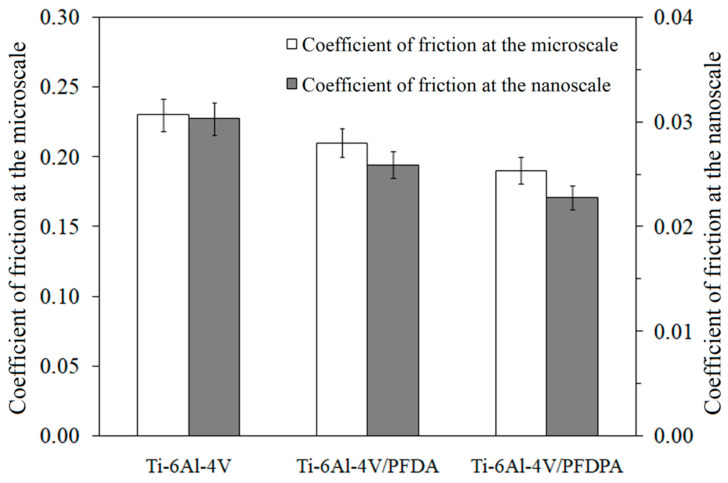
The coefficient of friction values obtained in nano- and millinewton load ranges for a silicon nitride counterbody.

In order to study the tribological behavior of the carboxylate and phosphonate layers created on the Ti-6Al-4V surface, the coefficient of friction, adhesive force, and wear rate were determined. Figure 7 shows the values of the coefficient of friction measured in the nano- and millinewton load ranges. 

In both cases, the coefficient of friction was always higher for a pure Ti-6Al-4V surface in comparison to the surfaces modified with SAMs. In the case of the tribological properties, fluorine in the tail group had a very big impact on the friction and wear. A significant reduction in the values of the coefficient of friction after the modification by fluoroalkyl compounds was connected with the hydrophobicity and low surface free energy of the modified surface. This was because, under low contact pressures, friction forces are dominated by surface interactions, mainly capillary forces. The adsorbed water molecules facilitate tribochemical reactions at the tribological interface and can cause the growth of friction [52]. The presence of hydrophobic self-assembled layers prepared by compounds containing fluorine prevents the formation of capillary films and tribochemical reactions. Out of both used components, the structure of Ti-6Al-4V/PFDPA exhibited better lubricating properties than those registered for Ti-6Al-4V/PFDA. This means that the SAMs of PFDPA showed lower friction compared to the SAMs of PFDA, both in nano- and millinewton load ranges. At the microscale, the use of PFDPA as a Ti-6Al-4V surface modifier allowed for reducing the coefficient of friction by about 17.4% compared to the unmodified Ti-6Al-4V, while the use of PFDA only reduced the friction by about 8.7%. The same trend was also observed at the nanoscale. Similar behavior was also recorded in the case of the adhesive force. The pure Ti-6Al-4V substrate showed a much higher adhesive force than the one with SAMs. The different values of adhesion between PFDPA and PFDA also suggest differences in the stability of the obtained layers. The head group of molecules had a significant impact on these parameters because it was responsible for forming the bond with the substrate. Therefore, sample Ti-6Al-4V/PFDPA with the phosphonic head group, which was characterized by the lowest surface energy and the highest water contact angle, showed the lowest adhesive force.

**Figure 8:**
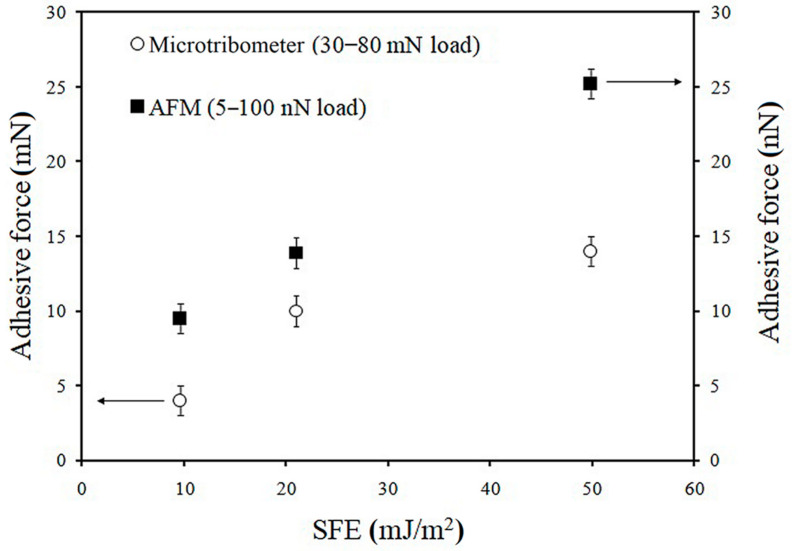
Adhesion force obtained for millinewton (microtribometer) and nanonewton (AFM) loads during tribological tests as a function of the SFE of investigated surfaces.

Figure 8 shows how the adhesive force changed as a function of the SFE. In the case of the AFM measurements, a linear correlation between the adhesive force and the surface energy was observed, as Bhushan pointed out [19]. Moreover, based upon a two-contact mechanics model, namely, the Johnson–Kendall–Roberts (JKR) model, it was also proven that the adhesive force (F) correlated with the surface energy (the dispersive and polar components) through the work of adhesion (W_A_):

F = 1.5πRW_A_, (3)

where R is the radius of the sphere (probe tip). 

As mentioned earlier, the considerable influence on the SFE value has a dispersive component, which is mainly contributed by Lifshitz–van der Waals (LW) forces [53]. The work of adhesion can be calculated using an analytical equation:

W_A_ = 2(γ_l_ γ_2_)^1/2^, (4)

where γ_l_ and γ_2_ are the dispersive components of the surface energy. 

Comparing the results shown in Table 1 and Figure 8, the acid–base component of the surface energy for the PFDPA and PFDA layers did not significantly influence the adhesion at the nano- and microscales, in contrast with the Lifshitz–van der Waals component. The lowest values of the SFE, the γ^LW^ component, and the adhesive force were obtained for Ti-6Al-4V modified with PFDPA. Comparing the values of adhesion for the nano- and millinewton load ranges for the carboxylate and phosphonate layer, the same trend was noticed (Figure 8). Unmodified substrates were characterized by the highest adhesion. As it is well known, when SAMs are disordered and hydrophilic, they easily adsorb water molecules, thus they exhibit a higher adhesive force. However, when SAMs are hydrophobic and ordered, they show low adhesion. After the modification, Ti-6Al-4V/PFDPA SAMs showed a lower adhesive force than Ti-6Al-4V/PFDA because its layers were more ordered and exhibited lower surface energy. 

It was found that the SFE (and both of its components) and also the adhesive force played important roles in affecting the tribological properties of the investigated surfaces. In the presented results, the adhesion force arose mainly from capillary forces [54,55]. The capillary force was reduced by creating a hydrophobic coating that prevented water accumulation. From the data presented above, it is clear that when an investigated surface exhibited the lowest values of SFE and adhesive force, it was characterized by the lowest coefficient of friction as well. 

The micro/nanoscale friction force (F_f_) is the sum of the adhesional component and friction force component consumed during the plastic deformation:

F_f_ = F_adh_ + F_def_, (5)

where F_adh_ is the adhesive force component consumed during shearing of the adhesive connections. The F_def_ force is a component that is dependent on plastic deformation and plowing. 

In the case of very small loads, at the nanoscale (nN), the friction force depends on the adhesive interactions in the vast majority of cases. Because the force associated with the consumption of the material was low, the friction force was practically entirely used for shearing the adhesive connections. At the nanoscale, the difference in the coefficient of friction values for PFDPA and PFDA also resulted from the pressure exerted by the tip, which more easily deformed for the less stable PFDA than the more stable PFDPA thin layer. Monolayer deformation during tip sliding on the surface is the reason for an increased shear force, and as a consequence, a higher friction force. In the millinewton load range, the lower coefficient of friction was exhibited by the surface with PFDPA, which was similar to the results obtained at the nanoscale. The trend in the contact angle and adhesive force results agreed well with the coefficient of friction data for the nano- and millinewton load ranges. In the case of the measurements at the nanoscale, the friction force was mainly related to the adhesive force but the friction force at the microscale also depended on the plastic deformation and plowing. Therefore, the lower values of the coefficient of friction were registered for tests conducted at the nanoscale in comparison to those obtained at the microscale (Figure 7).

**Figure 9:**
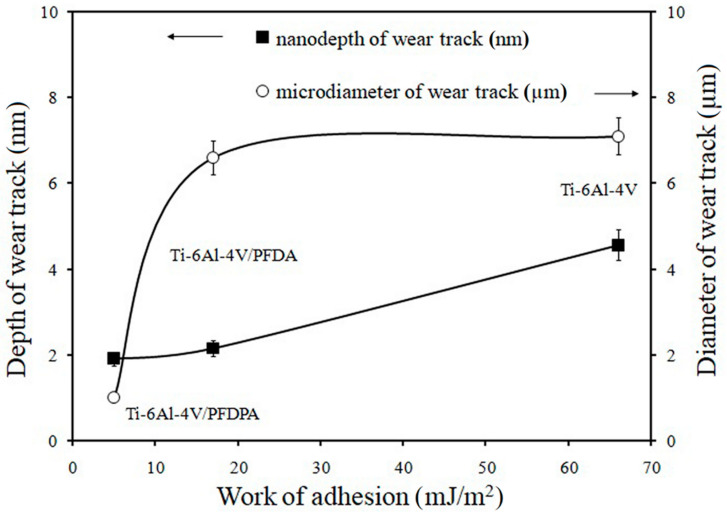
The relationship between the depth/diameter of the wear track and the work of adhesion.

**Figure 10:**
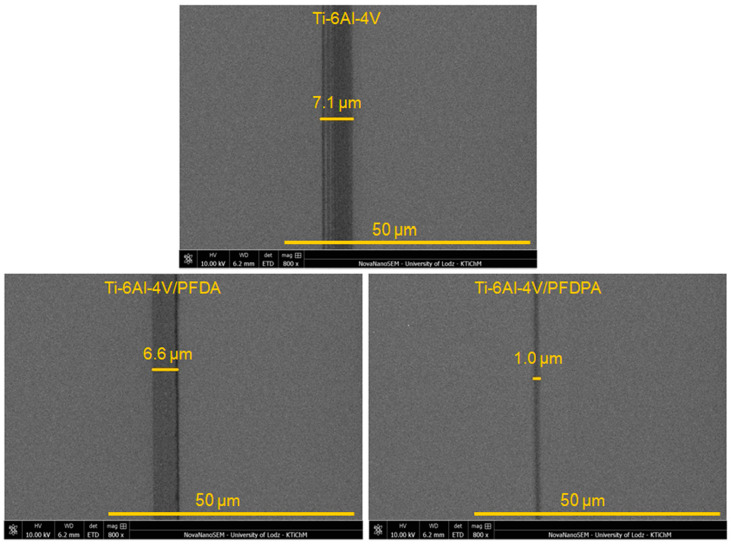
The images of the wear tracks after the tribology test at the microscale on the Ti-6Al-4V surface before and after modification.

The last factors that influenced the changes in the coefficient of friction were wear and the work of adhesion. As already mentioned earlier, the adhesive force correlated with the surface energy, while the SFE influenced the coefficient of friction and the work of adhesion. To check how the adhesion work affected the surface wear, this parameter was investigated for the Ti-6Al-4V surface before and after the deposition process. The correlation between the work of adhesion and the depth/diameter of the wear track results at the nano- and microscales are presented in Figure 9. Additionally, Figure 10 shows the microscopic images of the surfaces after the tribology tests at the microscale.

It was found that the pure Ti-6Al-4V exhibited a significantly higher work of adhesion in comparison with the PFDPA and PFDA layers. In this case, the presence of modifiers on the investigated surface caused a reduction in the work of adhesion. The work of adhesion results showed that after the deposition process of the hydrophobic layers, the depth/diameter of the wear track decreased (Figure 10). As we can see, the lowest value of this parameter was obtained for the PFDPA layer because it was the most hydrophobic and more stable and well-ordered in comparison with PFDA. Additionally, the phosphonic layer also exhibited excellent tribological performance, both at the nano- and microscales, as shown by the low values for the coefficient of friction and depth/diameter of the wear track.

## 4. Conclusions

The presented results of this investigation of phosphonate and carboxylate SAMs created on the oxidized surface of Ti-6Al-4V substrates show that due to the appropriate selection of parameters of the LPD process (in particular, in the case of the PFDA component), hydrophobic surfaces can be obtained. The results of the contact angle measurements indicated that the PFDPA component produced more ordered layers (forming monodentate binding with the Ti-6Al-4V surface) on the substrate than PFDA, which exhibited a low adhesive force and coefficient of friction at both the nano- and microscales. Each modified surface showed much lower adhesion and friction coefficients than the pure Ti-6Al-4V. Additionally, the correlation between the work of adhesion and the depth/diameter of the wear track was found, indicating that these parameters were significantly affected by the obtained tribological results. Surfaces with the CF_3_-terminated layers exhibited lower wear and work of adhesion in comparison to the unmodified Ti-6Al-4V surface. 

In summary, both of the fluoroalkyl compounds used for modification led to an increased hydrophobicity of the modified Ti-6Al-4V surface, decreased the values of the surface free energy, and improved the tribological properties. Therefore, the Ti-6Al-4V surface with the phosphonic acids may find applications as a protective layer where anti-frictional and anti-adhesion properties play a crucial role.

## Figures and Tables

**Table 1:** Surface free energy and its components for a system consisting of different SAMs created as a Ti-6Al-4V coating.

Materials	SFE (mJ/m^2^)	LW (mJ/m^2^)	AB (mJ/m^2^)
Ti-6Al-4V	49.9 ± 0.6	34.8 ± 0.2	12.1 ± 0.6
Ti-6Al-4V/PFDA	21.4 ± 0.3	19.9 ± 0.9	1.5 ± 0.1
Ti-6Al-4V/PFDPA	9.1 ± 0.3	8.1 ± 0.9	1.0 ± 0.1

SFE: surface free energy, LW: Lifshitz–van der Waals interaction, AB: acid–base interaction.
